# Airway, but not serum or urinary, levels of YKL-40 reflect inflammation in early cystic fibrosis lung disease

**DOI:** 10.1186/1471-2466-14-28

**Published:** 2014-02-27

**Authors:** Emmanuelle Fantino, Catherine L Gangell, Dominik Hartl, Peter D Sly

**Affiliations:** 1 The Queensland Children’s Medical Research Institute, The University of Queensland, Level 4, Foundation Building, Royal Children’s Hospital, Herston road, Herston, Brisbane, QLD 4059, Australia; 2 Department of Pediatrics, Section of Immunology and Infectious Diseases, University of Tubingen, Tubingen 72076, Germany

**Keywords:** Cystic fibrosis, YKL-40, Biomarker, Lung disease

## Abstract

**Background:**

Cystic fibrosis (CF) lung disease begins in early life and is progressive with the major risk factor being an exaggerated inflammatory response. Currently, assessment of neutrophilic inflammation in early cystic fibrosis (CF) lung disease relies on bronchoalveolar lavage (BAL). The chitinase-like protein YKL-40 is raised in sputum and serum of adults with CF. We investigated YKL-40 in BAL, serum and urine to determine whether this reflected inflammation and infection in young children with CF.

**Methods:**

YKL-40 was measured in matched samples of BAL, serum and urine obtained from 36 infants and young children with CF participating in an early surveillance program. Levels were compared to clinical data and markers of inflammation detected in the lung.

**Results:**

YKL-40 in BAL correlated with pulmonary infection [β=1.30 (SE 0.34), p < 0.001] and BAL markers of inflammation [macrophage number: r^2^ = 0.34, p < 0.001; neutrophil number: r^2^ = 0.74, p < 0.001; neutrophil elastase: r^2^ = 0.47, p < 0.001; CXCL8: r^2^ = 0.45, p < 0.001; IL-β: r^2^ = 0.62, p < 0.001]. YKL-40 was detectable in serum but levels did not correlate with BAL levels in the same individuals (r^2^ = 0.04, p = 0.14) or with inflammatory markers. YKL-40 was below the limit of detection in urine (30 pg/ml).

**Conclusions:**

This study demonstrates that levels of the chitinase-like protein YKL-40 reflect airway inflammation and infection in early CF lung disease. The lack of increased YKL-40 in serum in the absence of systemic inflammation limits the benefit of this potential biomarker in early disease.

## Background

Cystic fibrosis (CF) is still the most common life-limiting genetic disease in Caucasian populations. While survival and quality of life have improved in the past decades, the majority of children born with CF will still die from progressive lung disease in early to mid-adulthood. Lung disease may be present in infants within the first weeks of life [1-3] with abnormalities reported in: lung function [1,4]; pulmonary inflammation [2,5]; and infection, including with *Pseudomonas aerugino*sa [2,6]; and changes on chest computed tomography (CT) consistent with early onset bronchiectasis [2,3,7]. Once established, bronchiectasis is essentially irreversible [8] and progresses to respiratory failure and death or the need for lung transplantation in the majority of patients.

A growing body of evidence has demonstrated that the major risk factors for both the initiation and progression of bronchiectasis in early life include the presence of free neutrophil elastase (NE) [3], infection and inflammation [8]. The most reliable way of detecting pulmonary inflammation and infection in early life is by bronchoscopy and bronchoalveolar lavage (BAL) [9]. However, these procedures are invasive and cannot be repeated frequently.

To overcome the limitations inherent in BAL-based programs, biomarkers of infection and inflammation in the lung are urgently needed. A number of biomarkers have been proposed from studies in adults or older children with established CF lung disease. These include the chitinase-like protein YKL-40 [10], NE, tissue inhibitor of metalloproteinase-1 (TIMP-1) [11], and the elastin breakdown products desmosine and isodesmosine [12].

YKL-40 (chitinase-3-like-1; CHI3L1), also known as human cartilage glycoprotein 39 (HC gp-39), is a human glycoprotein that binds to chitin, but does not possess chitin hydrolase activity. YKL-40 is secreted by a variety of cells, including neutrophils [13] and macrophages [14] and expression is induced by IFN-γ [15] and release stimulated by IL-6 [16]. Although the exact function is unknown, it is associated with inflammation, extracellular tissue remodelling, fibrosis and solid carcinomas.

Serum YKL-40 has been investigated as a biomarker [17] for cancer [18], osteoarthritis [19], cardiovascular diseases [20] and a number of diseases involving inflammation, tissue remodelling and fibrosis [21,22]. YKL-40 levels have been also associated with asthma, atopy and other immune-related phenotypes [23,24]. Recently, increased YKL-40 levels in sputum were found to correlate with pulmonary function in adults with CF, suggesting YKL-40 usefulness as potential biomarker in CF lung disease [10]; increased levels were reported in sputum and, to a lesser extent, in serum from adults with CF compared to healthy control individuals. Thus, while not specific to CF, YKL-40 has the potential to be a biomarker of inflammation in early CF lung disease.

However, biomarkers that appear to be useful in established lung disease may not have the same value in early CF disease [25,26]. This situation is exemplified by the use of cyanide in respiratory secretions to indicate the presence of infection with *P. aeruginosa*. As *P. aeruginosa* is the only important cyanobacterium present in the lungs of patients with CF, the detection of cyanide in sputum or exhaled breath of patients with chronic *P. aeruginosa* infection [27,28] appeared to be a promising biomarker. However, when investigated in the BAL of infants with early lung disease the cyanide levels were more closely related to neutrophil number than to infection with *P. aeruginosa,* presumably related to production via neutrophil-derived thiocyanate [26].

The aim of the present study was to determine whether YKL-40 reflected inflammation in the lungs in early CF lung disease. We initially aimed to determine whether YKL-40 was present in the BAL of infants and young children with CF and whether YKL-40 levels related to markers of inflammation. We next aimed to determine whether YKL-40 could be detected in the serum and urine of the same children and whether YKL-40 could be used as a biomarker of inflammation in early CF lung disease.

## Methods

### Patient samples

Matched BAL, serum and urine samples were obtained from 36 infants and young children with CF who participated in the AREST CF early surveillance program (ESP). Details of the program and collection of biological samples have been described in detail elsewhere [2,3,7,8,29]. Briefly, participants diagnosed with CF via newborn screening were enrolled into the ESP. At three months of age, and yearly thereafter around the time of diagnosis, a series of procedures were performed including: BAL for detection of inflammation and infection; low dose CT scan for detection of structural abnormalities; and pulmonary function. At BAL, three aliquots of saline (1ml per kilogram of body weight) were instilled and retrieved from the right middle lobe. The first aliquot was used for microbiology analysis, and aliquots two and three were pooled for analyses of inflammation.

Methods for measuring cytokine concentrations, NE activity, cell counts and detection of infection have been described in detail previously (see online supplements in [2,8]). Briefly, IL-8 was measured using an ELISA (BD Opt EIA, BD Biosciences, San Diego, CA), other cytokines were measured using a standard cytometric bead array human inflammation kit (BD Biosciences, San Diego, CA), and free neutrophil elastase activity was measured using an enzymatic assay. Pulmonary infection was determined from BAL as previously described [30], with colony counts of specific organisms (excluding mixed oral flora) ≥ 10^5^ colony forming units /ml defined as pulmonary infection. However, the presence of *P. aeruginosa* in any density in BAL cultures was classed as infected.

The surveillance program was approved by the Princess Margaret Hospital for Children Ethics Committee (EC00270) of Perth, Australia (Approval number 1762/EP) and the Royal Children’s Hospital Human Research Ethics Committee (EC00238) of Melbourne, Australia (Approval number 25054), and conforms to the guidelines for conduct of research in children from the National Health and Medical Research Council, Australia. Informed consent was obtained from the parents of participants at the time of BAL for collection and use of participant samples.

Samples of BAL, serum and urine were drawn from the AREST CF biobank if aliquots that had been collected on the same occasion were available. Three groups of samples were obtained based on the presence of NE, and pulmonary infection with *P. aeruginosa* in BAL; Group 1: no NE and no infection; Group 2: NE present, no infection; Group 3: NE present, infection with *P. aeruginosa*. The subjects contributing specimens used in the present study were broadly representative of the entire AREST CF cohort.

### YKL-40

YKL-40 protein was measured in duplicate using a commercial Human Chitinase 3-like 1 Quantikine ELISA Kit Cat DC3L10 (R&D Systems, Inc. Minneapolis, MN, USA) in matched BAL, sera and urine samples stored at -80°C. The BAL and serum samples were diluted 1:500 and 1:50 in assay buffer. The urine samples were analysed neat. The linear range of the assay was 50-4000 pg/ml.

### C-Reactive protein (CRP)

CRP was measured in the same samples in duplicate using a commercial kit AlphaLISA C-Reactive Protein Research kit Cat AL233C AL233 C Lot No1658745 (PerkinElmer Inc. Waltham, Massachusetts, USA). The linear dynamic range was from 30 pg/ml to 400,000 pg/ml.

### Statistical analysis

Initially univariate analyses were conducted to determine effects of age, gender, meconium ileus, pancreatic sufficiency status, CF genotype and use of anti-staphylococcal prophylaxis (with Augmentin®) on YKL-40 measures to identify confounders. To account for the fact that individual patients could contribute more than one set of samples to the analyses, associations between YKL-40, in the BAL and serum, were compared with inflammatory markers, infection and bronchiectasis score using a random effects GLS regression analysis with a robust standard error by patient ID on logged data. Analyses were carried out using STATA, version 11 (StataCorp LP, College Station, TX, USA).

## Results

A total of 36 individual children contributed 55 unique annual visits. BAL fluid was available at 54 visits, serum at 51 visits and urine at 50 visits. The demographics and clinical features of the population are outlined in Table 1.

**Table 1:** Demographic and clinical characteristics

**Metric**	**Variable**	
Individual patient	Sex (m:f)	18:18
	Phe508del (homozygous: heterozygous)	20:16
	Pancreatic insufficient	33/36 (91.7%)
BAL^1^	Age at time of BAL (years) [mean ± SD]	3.95 ± 2.21
	Respiratory symptoms at time of BAL	22 (40%)
	Respiratory infection in BAL	41 (75%)
	*P. aeruginosa* infection in BAL	16 (29%)

^1^55 BAL were obtained from 36 patients, variables related to each BAL considered as a separate event.

YKL-40 was detected in BAL and serum, although levels in urine were below the limit of detection of the assay in all samples. Levels in BAL [group median (IQR): 34.33 (71.38) ng/ml] were higher than in serum [group median (IQR): 25.93 (16.78) ng/ml]. However, there were no associations between concentrations of YKL-40 in the BAL and YKL-40 in the serum using matched samples from the same individuals (r^2^ = 0.04, p = 0.138).

Levels of YKL-40 in BAL and serum were not associated with age at time of BAL, sex, initial presentation with meconium ileus or regular antibiotic prophylaxis (data not shown). Serum YKL-40, but not BAL YKL-40, was higher in those with pancreatic insufficiency (β(SE): 0.44(0.08), p < 0.001), although the association was weak. The number of children who were pancreatic insufficient was very high (91.7%); therefore, pancreatic sufficiency status was not included as a covariate in further analyses. Levels of YKL-40 in the BAL (p = 0.006), but not serum (p = 0.43), were significantly increased in those heterozygous for Phe508del compared to those homozygous for Phe508del. There were no associations between the presence of respiratory symptoms at the time of the BAL and YKL-40 levels in BAL or serum (Table 2).

**Table 2:** Associations between YKL-40 and pulmonary inflammation and infection

**Variable**	**YKL-40 in BAL**				**YKL-40 in serum**			
	**β**	**SE**	**r**^**2**^	**p**	**β**	**SE**	**r**^**2**^	**p**
BAL macrophages (×10^3^/ml fluid)	0.98	0.20	0.34	<0.001	0.10	0.11	0.02	0.34
BAL neutrophils (×10^3^/ml fluid)	0.58	0.07	0.74	<0.001	−0.003	0.04	0.0002	0.92
NE (ng/ml)	0.45	0.06	0.47	<0.001	−0.02	0.04	0.004	0.61
CXCL8 (pg/ml)	0.49	0.07	0.45	<0.001	−0.03	0.05	0.01	0.55
IL-1β (pg/ml)	0.64	0.06	0.62	<0.001	0.08	0.04	0.04	0.052
IL-6 (pg/ml)	0.40	0.21	0.14	0.060	−0.03	0.08	0.005	0.69
Respiratory symptoms at BAL	0.36	0.40	0.06	0.366	−0.10	0.17	0.005	0.57
Pulmonary infection								
• Any	1.30	0.34	0.17	<0.001	0.33	0.19	0.06	0.083
• *P. aeruginosa*	1.21	0.41	0.20	0.003	0.33	0.15	0.06	0.024

**Figure 1:**
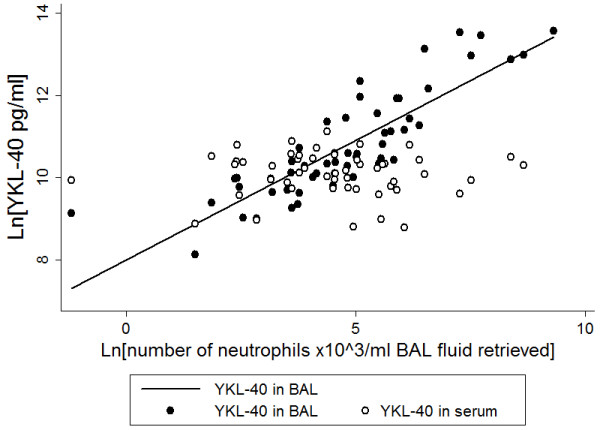
**Relationship of number of neutrophils in bronchoalveolar lavage (BAL) and YKL-40 in BAL (solid dots, solid line, r**^**2**^ **= 0.74, p < 0.001) and YKL-40 in serum (open dots, non-significant).**

**Figure 2:**
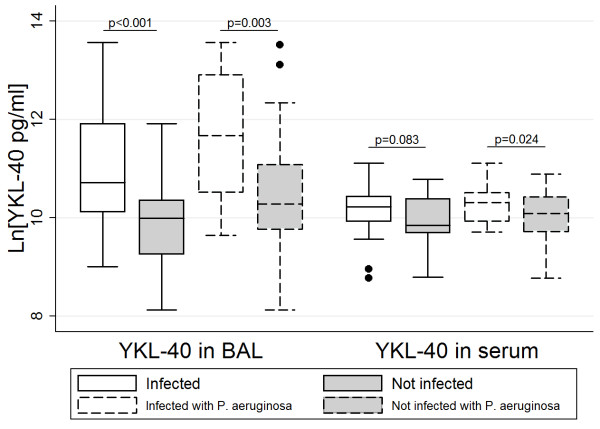
**YKL-40 concentrations in BAL and serum related to infection status, for those uninfected, infected with any organism or infected with *P aeruginosa*.** Note: the two infection groups are not mutually exclusive. Statistical comparisons are between each of the infected groups and the uninfected group. *Indicates the p-value of comparison between infected with *P. aeruginosa* and not infected with *P. aeruginosa.*

YKL-40 in BAL was significantly associated with most inflammatory markers in BAL including number of macrophages (r^2^ = 0.34, p < 0.001), number of neutrophils (r^2^ = 0.74, p < 0.001) (Figure 1), NE (r^2^ = 0.47, p < 0.001), CXCL8 (r^2^ = 0.45, p < 0.001) and IL-1β (r^2^ = 0.62, p < 0.001) (Table 2). Concentrations of YKL-40 in BAL were higher in the presence of any respiratory infection [β(SE): 1.30(0.34), p < 0.001] and with infection with *P. aeruginosa* [1.21(0.41), p = 0.003] (Figure 2). The associations between YKL-40 in BAL and number of neutrophils (p < 0.001) and IL-1β (p < 0.001) in BAL remained significant in a multivariate analysis.

In contrast to YKL-40 in BAL, YKL-40 measured in serum was not associated with pulmonary inflammation or with overall infection status (Table 2). There was a statistically significant, but weak (r^2^ = 0.06), association between serum YKL-40 and pulmonary infection with *P. aeruginosa* [0.33(0.15), p = 0.024] (Figure 2). Although this association was not present after inclusion in a multivariate model (p = 0.171). There were no associations between serum levels of YKL-40 and total or differential cell counts in blood (data not shown). Serum CRP was measured as an indication of systemic inflammation. Concentrations of CRP detected in this population were all below the clinically significant cut-off value of 5 mg/L [mean (sd): 0.07 (0.19) mg/L]. CRP levels were not associated with levels of YKL-40 measured in serum (data not shown).

## Discussion

The data from the present study demonstrate that concentrations of YKL-40 in BAL, but not in the serum, track with early indicators of lung disease in infants and young children with CF. Specifically YKL-40 in BAL reflects inflammation in the lungs, with positive associations seen with: the number of neutrophils and macrophages; concentration of NE; other markers of inflammation, such as IL-1β and CXCL8 concentrations; and with infection, in particular with *P. aeruginosa.* However, while YKL-40 was detectable in the serum of children in CF, the serum levels did not correlate with the BAL levels in the same patient nor with pulmonary inflammation. YKL-40 was not detectable in urine. Collectively, these findings indicate that YKL-40 in the serum and urine do not reflect pulmonary infection and inflammation, and therefore are not useful as a biomarkers of early stage CF lung disease.

The positive associations between BAL levels of YKL-40 and indicators of active neutrophilic inflammation in the lungs seen in the present study do suggest that YKL-40 is likely to be of neutrophilic origin in early CF lung disease, as suggested by previous *in vitro* cellular studies [13]. However, in contrast to previous reports in adults with CF [10], the results of the present study show that YKL-40 concentrations in serum did not correlate with concentrations in BAL or with other markers of pulmonary inflammation, infection or structural lung disease. This difference between children and adults may be due to the different stage of pulmonary disease, particularly the severity of neutrophilic airway inflammation, which increases continuously from infant to adult CF lung disease.

We did confirm the lack of systemic inflammation by low CRP levels in the serum of children in the present study. In the study by Hector *et al.,* it is possible that either YKL-40 generated in the lungs “spilled over” into the serum of their patients or that the serum levels reflected systemic inflammation. Indeed other studies have demonstrated increased levels of YKL-40 in the blood in association with severe inflammation and sepsis [31]. However, neither systemic inflammation, nor quantities of YKL-40 “spilling over” into the serum appears to be operative in the young children with CF we studied.

Association between serum levels of YKL-40 and the presence of *P. aeruginosa* infection was weak but significant, although when included in a multivariate model the association was no longer significant. YKL-40 is a marker of inflammation in both infectious and non-infectious diseases, although due to associations with inflammation and YKL-40 levels in non-infectious disease, it is likely YKL-40 is a reflection of inflammation rather than bacterial load [18-20]. We have previously reported increased inflammation with *P. aeruginosa* infection in the lungs of paediatric patients with CF [30], although other studies have reported no correlations between lung and serum cytokine levels in CF, even those chronically infected with *P. aeruginosa*[32,33]. However, the children in the present study do not have chronic *P. aeruginosa* infection and increased YKL-40 or other inflammatory markers in the blood of these patients due to *P. aeruginosa* infection seems unlikely. This is verified as addition of the *P. aeruginosa* into a multivariate model reduced the significant associations between *P. aeruginosa* and YKL-40 levels in the serum.

We must caution that the sample size of the current study is small, however, the inability to detect YKL-40 in urine and the lack of associations between serum levels of YKL-40 with those in BAL or with markers of pulmonary inflammation in early life seriously question whether larger studies are warranted in this age group.

While bronchoscopy and CT scans are used to detect early changes in lung disease, these procedures cannot be performed frequently and can only be performed when children are well enough to undergo general anaesthesia. Biomarkers to identify early stage disease in CF are important to monitor and track disease progression in the hope to stem progression. The AREST CF program is ideally suited to the discovery and validation of biomarkers of early lung disease. The simultaneous collection and storage of BAL, serum and urine at 3 months of age and at annual reviews until 6 years of age, together with extensive characterization of clinical physiological and radiological aspects of early lung disease provide unique opportunities to examine the correlation of proposed biomarkers. The group has previously reported that serum antibodies against *P. aeruginosa* were not predictive of *P. aeruginosa* grown in BAL [25] and that the presence of cyanide in BAL was not predictive of *P. aeruginosa* infection in early life [26]. The results of the present study further demonstrate that biomarkers that show promise in following CF disease severity in older children and adults with established lung disease must be validated in early life before being adopted.

## Conclusions

In summary, while YKL-40 in BAL is reflective of inflammation in early CF lung disease, it is not increased in serum or urine in this age group and does not seem to offer an advantage over measuring neutrophilic inflammation and NE activity directly. Further studies are required to identify the pathophysiological role of the chitinase-like protein in the pulmonary microenvironment in CF lung disease.

## Abbreviations

AREST CF: Australian respiratory early surveillance team for cystic fibrosis; BAL: Bronchoalveolar lavage; CHI3L1: Chitinase-3-like-1; CF: Cystic fibrosis; CRP: C-reactive protein; CT: Computed tomography; CXCL8: Chemokine (C-X-C motif) ligand 8; ELISA: Enzyme-linked immune sorbent assay; ESP: Early surveillance program; HCgp-39: Human cartilage glycoprotein 39; IL-: Interleukin-; INF-: Interferon-; NE: Neutrophil elastase; TIMP-1: Tissue inhibitor of metalloproteinase-1; YKL-40: A 40 kilodalton chitinase-like protein named after the first three N-terminal amino acids, tyrosine (Y), lysine (K) and leucine (L).

## Competing interests

The authors declare that they have no competing interests.

## Authors’ contributions

Conception and design PS, EF, DH. Analysis and interpretation: All authors. Drafting the manuscript: EF, CG, PS. All authors read and approved the final manuscript.

## Pre-publication history

The pre-publication history for this paper can be accessed here:

http://www.biomedcentral.com/1471-2466/14/28/prepub
